# Synopses

**DOI:** 10.1080/13577149878091

**Published:** 1998-03


					Sarcoma (1998) 2, 1 2
SYNOPSES
Dr Martin H. Robinson
Dr Robinson is Senior Lecturer in Clinical Oncology, in the Section of Clinical Oncology, at Weston
Park Hospital, Shef(R) eld, UK. He has been involved in the (R) eld of sarcomas for 10 years and runs a
sarcoma clinic at the above hospital. His other main area of interest is the treatment of head and neck
cancer. He is chairman of the Local Treatment Group of the EORTC Soft Tissue and Bone Sarcoma
Group and is on the steering committee of the MRC Soft Tissue Sarcoma Group. He is also chairman
of the North Trent Sarcoma Group. As main editor of Sarcoma his aims are to improve multidisciplinary
investigations, treatment and research into sarcomas.
1357-714 X/98/010001 02 O 1998 Carfax Publishing Ltd
2 Synopses
Dr Raphael E. Pollock
Dr Pollock is Head, Division of Surgery, Professor and Chairman, Department of Surgical Oncology,
and also holds a joint appointment in the Department of Tumor Biology, all at the University of Texas
M. D. Anderson Cancer Center, Houston, Texas. Dr Pollock is a member of eleven editorial boards,
and is the Sarcoma Section Editor for the Annals of Surgical Oncology, and the North American Editor
for the journal Sarcoma. Dr Pollock also chairs the American College of Surgeon Commission on
Cancer National Sarcoma Committee, is a member of the Board of Directors of the Connective Tissue
Oncology Society, and Co-Chairman of the Sarcoma Committee of the National Cancer Center
Coalition. Dr Pollock is the principal investigator of an NIH funded laboratory research program that
is examining the control mechanisms underlying soft tissue sarcoma proliferation and dissemination
focusing on the role of p53 tumor suppressor gene mutations in soft tissue sarcoma (RO1 CA 67802;
Therapy and biological correlates of soft tissue sarcoma' ).

				

